# Tomatidine Improves Pulmonary Inflammation in Mice with Acute Lung Injury

**DOI:** 10.1155/2021/4544294

**Published:** 2021-09-07

**Authors:** Wen-Chung Huang, Shu-Ju Wu, Ya-Ling Chen, Chwan-Fwu Lin, Chian-Jiun Liou

**Affiliations:** ^1^Graduate Institute of Health Industry Technology, Research Center for Chinese Herbal Medicine, Chang Gung University of Science and Technology, Taoyuan City 33303, Taiwan; ^2^Division of Allergy, Asthma, and Rheumatology, Department of Pediatrics, Chang Gung Memorial Hospital, Linkou, Taoyuan City 33303, Taiwan; ^3^Department of Nutrition and Health Sciences, Chang Gung University of Science and Technology, Taoyuan City 33303, Taiwan; ^4^Aesthetic Medical Center, Department of Dermatology, Chang Gung Memorial Hospital, Linkou, Guishan Dist, Taoyuan 33303, Taiwan; ^5^School of Nutrition and Health Sciences, Taipei Medical University, 250 Wu-Hsing Street, Taipei City 11031, Taiwan; ^6^Department of Cosmetic Science, Research Center for Chinese Herbal Medicine, Chang Gung University of Science and Technology, Taoyuan City 33303, Taiwan; ^7^Department of Anesthesiology, Chang Gung Memorial Hospital, Linkou, Guishan Dist, Taoyuan City 33303, Taiwan; ^8^Department of Nursing, Division of Basic Medical Sciences, Research Center for Food and Cosmetic Safety, Chang Gung University of Science and Technology, Taoyuan City 33303, Taiwan

## Abstract

Tomatidine, which is isolated from green tomato, can ameliorate inflammation and oxidative stress in cells and animal experiments and has been shown to improve airway inflammation in a murine model of asthma. Here, we investigated whether tomatidine can ameliorate acute lung injury in mice. Mice were given tomatidine by intraperitoneal injection for 7 consecutive days, and then, lung injury was induced via intratracheal instillation of lipopolysaccharide (LPS). Tomatidine reduced inflammatory cytokine expressions in bronchoalveolar lavage fluid (BALF), attenuated neutrophil infiltration in the BALF and lung tissue, increased superoxide dismutase activity and glutathione levels, and alleviated myeloperoxidase expression in the lung tissue of mice with lung injury. Tomatidine also decreased inflammatory cytokine and chemokine gene expression in inflammatory lungs and attenuated the phosphorylation of mitogen-activated protein kinase and nuclear factor kappa B. Furthermore, tomatidine enhanced the production of heme oxygenase-1, decreased the secretion of inflammatory cytokines and chemokines in LPS-stimulated lung epithelial cells, and attenuated THP-1 monocyte adhesion. Our findings suggest that tomatidine attenuates oxidative stress and inflammation, improving acute lung injury in mice.

## 1. Introduction

Acute lung injury (ALI) is a serious respiratory disease that can cause severe clinical complications and high mortality and can also lead to acute respiratory distress syndrome [1]. The main clinical features of ALI are excessive lung inflammation and neutrophil infiltration of the lungs, as well as excessive inflammatory cytokine and chemokine secretions, which induce lung cell damage, causing severe diffuse pulmonary infiltrates, increased vascular permeability, pulmonary edema, and reduced respiratory gas exchange [2, 3]. Therefore, patients with ALI have difficulty breathing, which can eventually lead to respiratory failure and death [4].

Environmental factors and irritants can induce ALI, including cigarette smoke, air pollution, and bacterial infections [4, 5]. Lipopolysaccharide (LPS) is a biologically active molecule found in the cell wall of Gram-negative bacteria. An innate immune response can be activated by LPS to resist the invasion of microorganisms [6, 7]. *Pseudomonas aeruginosa*-infected airways have also been used as a model of lung injury in mice [8]. Induction of acute lung inflammation by intratracheal administration of LPS is a common animal model for studying the pathological mechanism of ALI [6]. Toll-like receptor 4 (TLR4) is the specific binding ligand for LPS. When the lungs are infected with bacteria, LPS stimulates TLR4 activation and inflammatory mediator overproduction mainly through the activation of the MYD88-dependent TLR4 pathway, leading to lung damage and ALI development [7]. The inflammatory signals induced by LPS also cause nuclear factor kappa B (NF-*κ*B) activation, inducing inflammatory mediators and cytokine productions [9]. In addition, mitogen-activated protein kinase (MAPK) contributes to the activation of inflammatory-associated genes in the lungs [2]. Therefore, attenuating MAPK and NF-*κ*B is an effective strategy to alleviating the inflammatory response in ALI.

Tomatidine is isolated from the immature tomato fruit [10] and has been shown to induce apoptosis and attenuate the proliferation of lung cancer cells [11]. A previous study found that tomatidine could reduce the expressions of inflammatory mediator in LPS-stimulated macrophages [12]. We have also found that tomatidine can suppress mucin production, airway inflammation, and airway hyperresponsiveness in ovalbumin-induced asthma mouse model through suppression of Th2 cell activity [13]. Therefore, we evaluated whether tomatidine alleviates acute lung injury and investigated the molecular mechanisms underlying inflammation and oxidative stress in mice.

## 2. Materials and Methods

### 2.1. Materials

Tomatidine was purchased from Sigma-Aldrich (St. Louis, MO, USA). For animal experiments, tomatidine was dissolved in dimethyl sulfoxide (DMSO), and the working solution was formulated as 5 mg/kg/50 *μ*l and 10 mg/kg/50 *μ*l. For cell experiments, tomatidine was dissolved in DMSO solution at a concentration of 100 mM. DMSO was ≤0.1% in culture medium as described previously [14].

### 2.2. Animal Model Preparation

Male BALB/c mice (aged 6–8 weeks) were purchased from National Laboratory Animal Center (Taipei, Taiwan), and their use was approved by the Animal Care and Use Committee of Chang Gung University of Science and Technology (IUCUC 2016-002). All mice were maintained in a standard animal housing and fed with a standard chow diet and clean water. Mice were randomly assigned to four groups (*n* = 8 mice each): normal control mice (N group), treatment with LPS, and LPS-induced mice given 5 mg/kg or 10 mg/kg of tomatidine (T5 and T10 groups, respectively). From day 1 to day 7, mice were intraperitoneally injected with DMSO (N and LPS groups) or tomatidine (T5 and T10 groups). Next, mice received 50 *μ*l LPS solution (containing 50 *μ*g LPS in 50 *μ*l PBS) or normal saline intratracheally on day 8 for 4 hours. Finally, the mice were anesthetized and sacrificed to collect the BALF and lung tissue. Blood was also harvested and centrifuged to collect the serum and stored at −80°C.

### 2.3. Neutrophil Numbers and Protein Concentration in the BALF

Mice were anesthetized and intubated by an indwelling needle into the trachea. Normal saline was used to wash the respiratory tract and lungs three times to collect the fluid that was defined as BALF [15]. BALF was used to detect cytokine and chemokine levels, and neutrophils were stained and calculated using Giemsa stain solution under the optical microscope (Olympus, Tokyo, Japan).

### 2.4. Lung Wet/Dry Weight Ratio

The right lung was collected and weighed as wet weight (*W*). The lung tissue was placed in the oven at 80°C for 48 h to obtain the dry weight (*D*). The lung *W*/*D* ratio was used to evaluate lung edema.

### 2.5. Histological Analysis

Lung tissues were fixed and embedded in paraffin. The samples were cut into 6 *μ*m sections and then stained with hematoxylin and eosin (H&E). Lastly, neutrophil infiltration was observed under an optical microscope (Olympus).

### 2.6. Malondialdehyde, Glutathione, and Superoxide Dismutase

Lung tissues were homogenized, and malondialdehyde (MDA) activity was assayed using the MDA assay kit (Sigma) [16]. Furthermore, glutathione (GSH) levels and SOD activity were examined using a GSH assay kit and SOD determination kit (Sigma), respectively.

### 2.7. Myeloperoxidase Activity

Lung tissues were homogenized and collected. The myeloperoxidase (MPO) assay kit (Sigma) was used to assay MPO activity with a microplate spectrophotometer (BioTek, Bedfordshire, United Kingdom).

### 2.8. Real-Time PCR Analysis

Lung RNA was extracted using TRIzol reagent. cDNA was synthesized, and specific genes were labeled using the singleplex SYBR Green system (Bio-Rad, CA, USA) to investigate gene expression on the iCycler Real-Time PCR System (Bio-Rad), including CCL5, COX-2, IL-1*β*, IL-6, iNOS, ICAM-1, MCP-1, and TNF-*α*. The reaction condition included predenaturation at 95°C for 10 min, gene amplification in 40 cycles of 95°C for 15 seconds, annealing at 60°C for 1 minute, and extension at 72°C for 1 min as described previously [15].

### 2.9. Western Blot

Proteins were separated by SDS-PAGE, transferred to PVDF membranes, and incubated with specific antibodies, including I*κ*B-*α*, lamin B1, Nrf2, HO-1, and phosphorylated-I*κ*B-*α* (Santa Cruz, CA, USA), phosphorylated-p38, phosphorylated-ERK 1/2, phosphorylated-JNK, and *β*-actin (Sigma), and p38, ERK1/2, COX-2, JNK, and ICAM-1 (Cell Signaling Technology, MA, USA). Next, the membranes were washed and treated with antibody-HRP conjugates and the protein signals were detected using an enhanced chemiluminescence solution. Images were obtained by a BioSpectrum 600 system (UVP, Upland, CA, USA).

### 2.10. Tomatidine Treatment of A549 Cells

Tomatidine was dissolved in DMSO solution (≤0.1% for all cell experiments). A549 cells were maintained in F-12K medium and treated with 0-10 *μ*M tomatidine for 1 h. The cells were then incubated with 1 *μ*g/ml LPS for 24 h. The supernatants were collected to detect specific chemokines or cytokines by ELISA.

### 2.11. ELISA

Cell culture medium, serum, and BALF were collected to detect the levels of CCL5, ICAM-1, IL-1*β*, IL-8, IL-6, MCP-1, and TNF-*α* using sandwich ELISA kits (R&D, Minneapolis, MN, USA) as described previously [17]. Using a microplate reader, we detected specific protein concentrations at an optical density (OD) of 450 nm.

### 2.12. Cell-Cell Adhesion

A549 cells were treated with tomatidine and then stimulated with LPS. THP-1 cells were incubated with calcein-AM (Sigma). Subsequently, the A549 and THP-1 cells (green fluorescent) were cocultured to assay THP-1 adhesion by fluorescence microscopy (Olympus) [18, 19].

### 2.13. Statistical Analysis

All data are expressed as the means ± standard error of the mean (SEM), based on at least three independent experiments. Experimental data were analyzed using ANOVA, followed by Dunnett's post hoc test. *p* < 0.05 was considered significant.

## 3. Results

### 3.1. Tomatidine Decreases Neutrophil Infiltration and Inflammation

**Figure 1:**
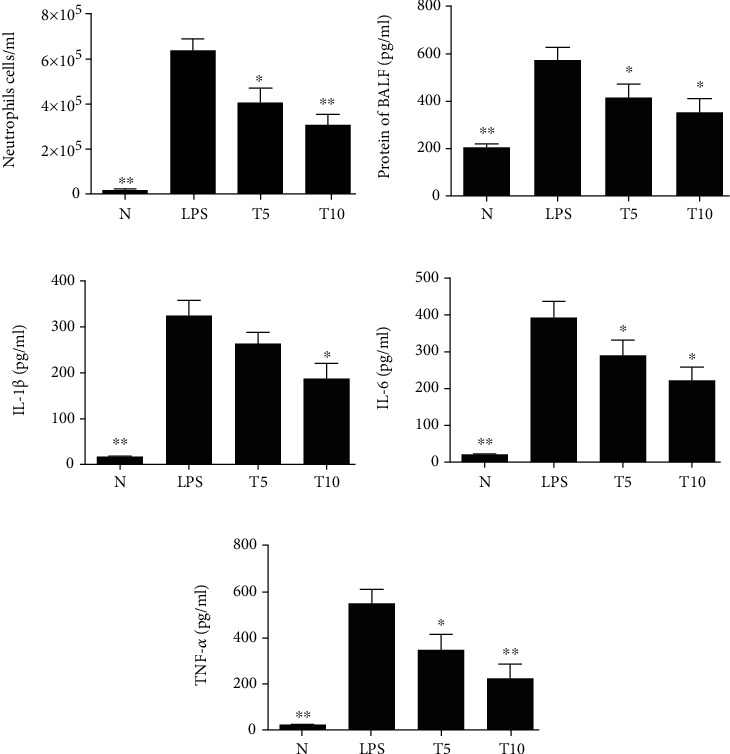
Tomatidine alleviated LPS-induced lung injury. Tomatidine affected (a) neutrophil numbers and (b) protein concentrations in the BALF. Tomatidine reduced (c) IL-1*β*, (d) IL-6, and (e) TNF-*α* concentrations in the BALF. Data are presented as the mean ± SEM. ^∗^*p* < 0.05 and ^∗∗^*p* < 0.01 vs. LPS group. N group: normal control mice; LPS group: LPS treatment only; T5 group: 5 mg/kg tomatidine plus LPS; T10 group: 10 mg/kg tomatidine plus LPS.

**Figure 2:**
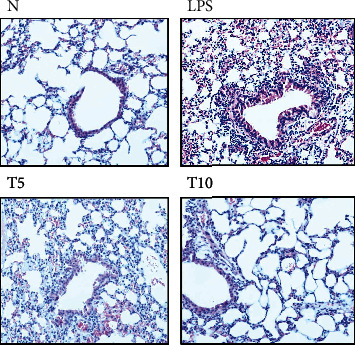
Tomatidine reduces neutrophil infiltration in lung tissue. HE staining (×200 magnification) of lung tissue to observe neutrophil infiltration. N group: normal control mice; LPS group: LPS treatment only; T5 group: 5 mg/kg tomatidine plus LPS; T10 group: 10 mg/kg tomatidine plus LPS.

Tomatidine effectively decreased neutrophil numbers in the BALF compared to LPS (T5: 4.1 × 10^5^ ± 6.4 × 10^4^ cells/ml, *p* < 0.05; T10: 3.1 × 10^5^ ± 4.8 × 10^4^ cells/ml, *p* < 0.01 vs. LPS: 6.4 × 10^5^ ± 5.0 × 10^4^ cells/ml) (Figure 1(a)). Moreover, the concentrations of total protein, IL-1*β*, IL-6, and TNF-*α* in BALF were reduced in the T5 and T10 groups (IL-1*β*, T5: 263.4 ± 24.64 pg/ml, *p* = 0.21; T10: 186.3 ± 34.96 pg/ml, *p* < 0.05 vs. LPS: 325.00 ± 33.35 pg/ml) (IL-6, T5: 290.20 ± 42.36 pg/ml, *p* < 0.05; T10: 222.11 ± 36.02 pg/ml, *p* < 0.05 vs. LPS: 392.80 ± 44.52 pg/ml) (TNF-*α*, T5: 352.05 ± 64.39 pg/ml, *p* < 0.05; T10: 226.52 ± 60.89 pg/ml, *p* < 0.01 vs. LPS: 551.02 ± 62.19 pg/ml) (Figures 1(b)–1(e)). In lung biopsy sections, neutrophil infiltration was ameliorated when mice with LPS-induced lung injury were treated with tomatidine (Figure 2).

### 3.2. Tomatidine Regulated SOD, GSH, and MDA Activity in the Lungs

**Figure 3:**
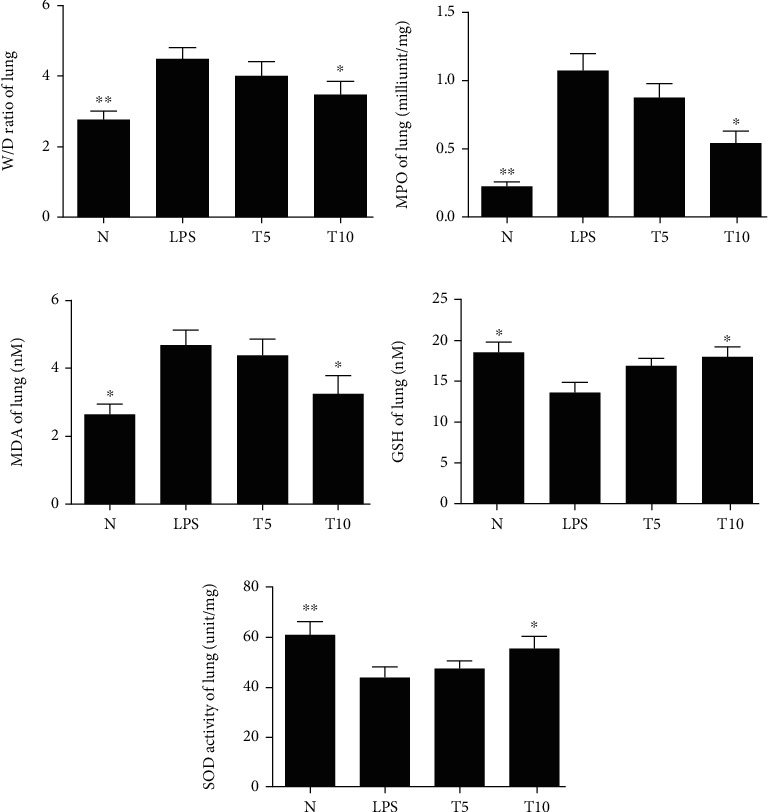
Tomatidine alleviated oxidative stress in LPS-induced lung injury. In the lungs, tomatidine regulated (a) the *W*/*D* ratio, (b) the MPO, (c) MDA, (d) GSH levels, and (e) SOD activity. Data are presented as the mean ± SEM. ^∗^*p* < 0.05 and ^∗∗^*p* < 0.01 vs. LPS group. N group: normal control mice; LPS group: LPS treatment only; T5 group: 5 mg/kg tomatidine plus LPS; T10 group: 10 mg/kg tomatidine plus LPS.

Tomatidine treatment effectively decreased the *W*/*D* ratios in mice with lung injury (T5: 4.11 ± 0.42, *p* = 0.37; T10: 3.49 ± 0.37, *p* < 0.05 vs. LPS: 4.49 ± 0.33) (Figure 3(a)). Tomatidine also alleviated MOP and MDA activity and promoted GSH and SOD expression in mice with lung injury (MPO, T5: 0.88 ± 0.10 mU/mg, *p* = 0.24; T10: 0.54 ± 0.09 mU/mg, *p* < 0.05 vs. LPS: 1.07 ± 0.13 mU/mg) (MDA, T5: 4.41 ± 0.45 nM, *p* = 0.67; T10: 3.27.5 ± 0.52 nM, *p* < 0.05 vs. LPS: 4.69 ± 0.45 nM) (GSH, T5: 16.98 ± 0.87 nM, *p* = 0.07; T10: 18.01 ± 1.24 nM, *p* < 0.05 vs. LPS: 13.68 ± 1.22 nM) (SOD, T5: 47.51 ± 3.21 U/mg, *p* = 0.67; T10: 55.50 ± 4.99 U/mg, *p* < 0.05 vs. LPS: 44.01 ± 4.26 U/mg) (Figures 3(b)–3(e)).

### 3.3. Tomatidine Suppressed Inflammatory Mediators in Lung Tissue

**Figure 4:**
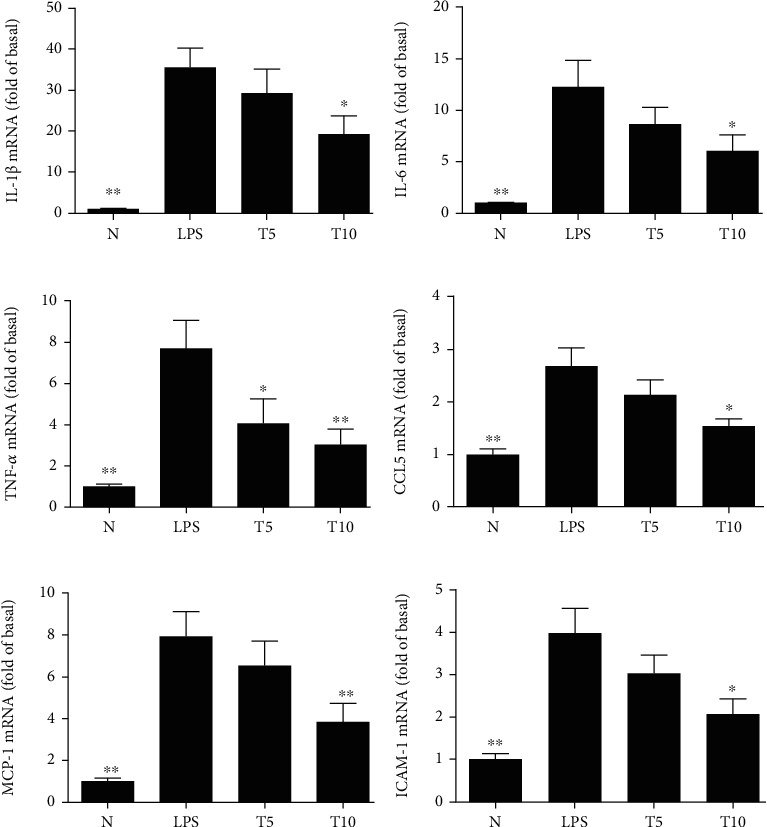
Tomatidine modulated gene expression in lung tissue. (a) IL-1*β*, (b) IL-6, (c) TNF-*α*, (d) CCL5, (e) MCP-1, and (f) ICAM-1. Fold expression is shown relative to *β*-actin expression. Data are presented as the mean ± SEM. ^∗^*p* < 0.05 and ^∗∗^*p* < 0.01 vs. LPS group. N group: normal control mice; LPS group: LPS treatment only; T5 group: 5 mg/kg tomatidine plus LPS; T10 group: 10 mg/kg tomatidine plus LPS.

**Figure 5:**
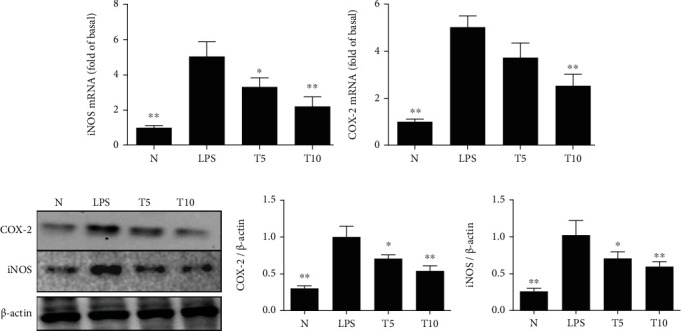
Tomatidine inhibited inflammatory mediators in mice. Tomatidine decreased the (a) gene expression of iNOS and (b) COX-2 in the lungs and suppressed (c) iNOS and COX-2 protein expressions. (d) The fold change in the expressions of COX-2 and iNOS protein was measured relative to the expression of *β*-actin, respectively. Data are presented as the mean ± SEM. ^∗^*p* < 0.05 and ^∗∗^*p* < 0.01 vs. LPS group. N group: normal control mice; LPS group: LPS treatment only; T5 group: 5 mg/kg tomatidine plus LPS; T10 group: 10 mg/kg tomatidine plus LPS.

**Figure 6:**
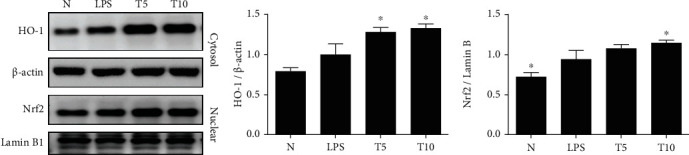
Tomatidine regulated HO-1 expression in the lung of mice. (a) Tomatidine increased HO-1 and Nrf2 protein expression. (b) The fold change in the expression of HO-1 and Nrf2 protein was measured relative to the expression of *β*-actin and lamin B, respectively. Data are presented as the mean ± SEM. ^∗^*p* < 0.05 vs. LPS group. N group: normal control mice; LPS group: LPS treatment only; T5 group: 5 mg/kg tomatidine plus LPS; T10 group: 10 mg/kg tomatidine plus LPS.

Tomatidine effectively decreased the gene expression of IL-1*β*, IL-6, TNF-*α*, CCL5, MCP-1, and ICAM-1 (Figure 4). Tomatidine also decreased gene and protein expression of iNOS and COX-2 (Figure 5). Furthermore, increased HO-1 expression in the cytoplasm and Nrf2 production in the nucleus were observed in the T5 and T10 groups (Figure 6).

### 3.4. Tomatidine Regulated NF-*κ*B and MAPK Signals

**Figure 7:**
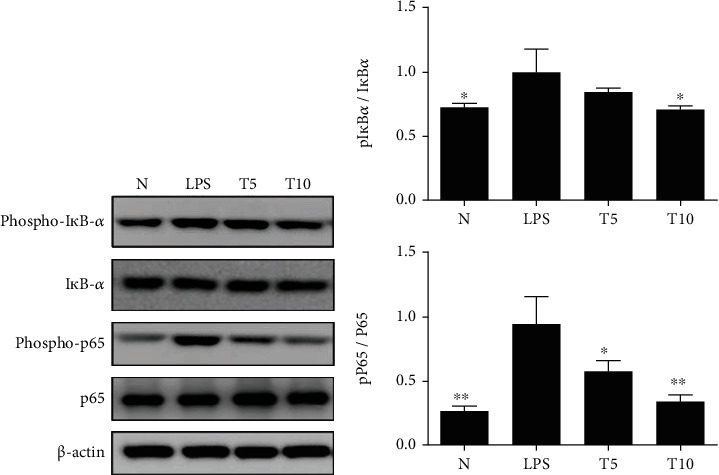
Tomatidine regulated NF-*κ*B pathway in the lung of mice. (a) Tomatidine increased I*κ*B-*α* and p65 phosphorylation in the lung. (b) The fold change in the expression of phosphorylated I*κ*B*α* and phosphorylated p65 protein was measured relative to the expression of I*κ*B*α* and p65, respectively. Data are presented as the mean ± SEM. ^∗^*p* < 0.05 and ^∗∗^*p* < 0.01 vs. LPS group. N group: normal control mice; LPS group: LPS treatment only; T5 group: 5 mg/kg tomatidine plus LPS; T10 group: 10 mg/kg tomatidine plus LPS.

**Figure 8:**
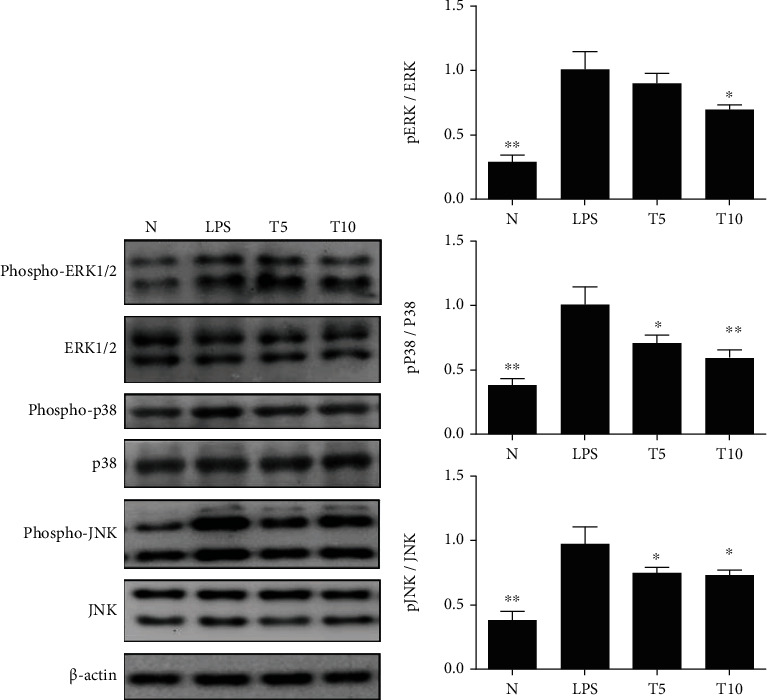
Tomatidine regulated the MAPK pathway in the lung of mice. (a) Tomatidine increased phosphorylation of ERK1/2, p38, and JNK protein expression. (b) The fold change in the expression of phosphorylated ERK1/2, p38, and JNK protein was measured relative to the expression of ERK1/2, p38, and JNK, respectively. Data are presented as the mean ± SEM. ^∗^*p* < 0.05 and ^∗∗^*p* < 0.01 vs. LPS group. N group: normal control mice; LPS group: LPS treatment only; T5 group: 5 mg/kg tomatidine plus LPS; T10 group: 10 mg/kg tomatidine plus LPS.

**Figure 9:**
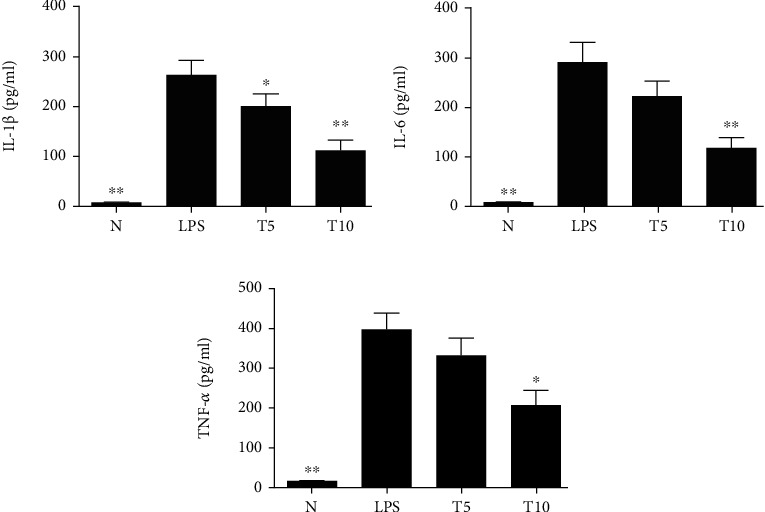
Tomatidine modulated serum cytokine levels, including (a) IL-1*β*, (b) IL-6, and (c) TNF-*α*, in ALI mice. Data are presented as the mean ± SEM. ^∗^*p* < 0.05 and ^∗∗^*p* < 0.01 vs. LPS group. N group: normal control mice; LPS group: LPS treatment only; T5 group: 5 mg/kg tomatidine plus LPS; T10 group: 10 mg/kg tomatidine plus LPS.

Tomatidine reduced I*κ*B-*α* and p65 phosphorylation in injured lung tissue (Figure 7) and alleviated ERK1/2, p38, and JNK phosphorylation (Figure 8). Tomatidine treatment also attenuated the serum concentration of IL-1*β*, IL-6, and TNF-*α* (Figure 9).

### 3.5. Tomatidine Suppressed Inflammation in LPS-Activated A549 Cells

**Figure 10:**
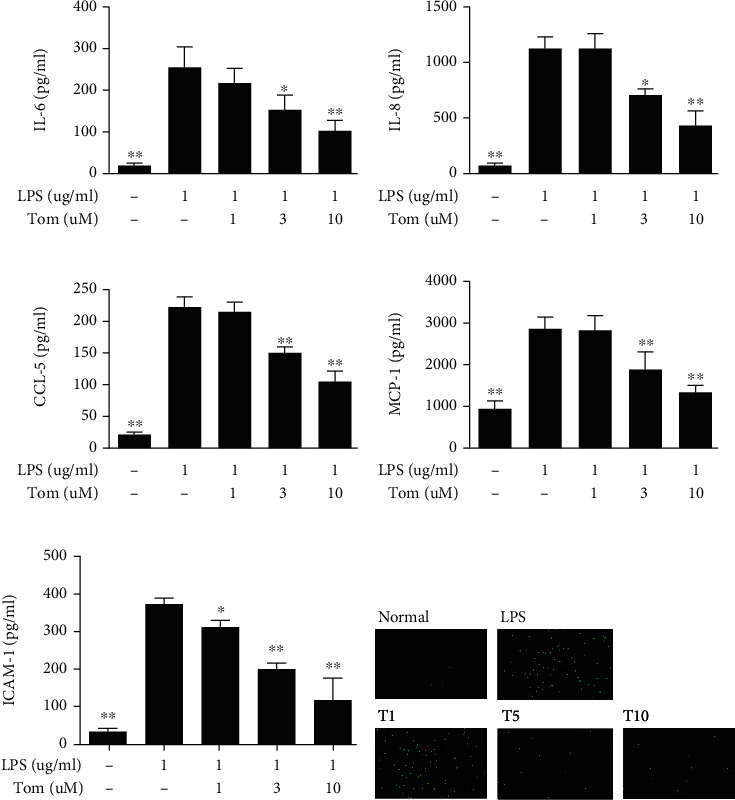
Tomatidine affects inflammation in LPS-stimulated A549 cells. Concentration of (a) IL-6, (b) IL-8, (c) CCL5, (d) MCP-1, and (e) ICAM-1 detected by ELISA. (f) Tomatidine (1-10 *μ*M) inhibited THP-1 monocyte adherence to active A549 cells. Data are presented as the mean ± SEM. ^∗^*p* < 0.05 and ^∗∗^*p* < 0.01 vs. LPS group.

Tomatidine dose-dependently reduced IL-6, IL-8, MCP-1, and CCL5 expression compared to LPS-activated A549 cells (Figures 10(a)–10(d)). In addition, tomatidine inhibited ICAM-1 secretion (Figure 10(e)) and reduced THP-1 cell adherence to LPS-stimulated A549 cells (Figure 10(f)).

## 4. Discussion

Tomatidine is thought to attenuate the inflammatory response in LPS-induced macrophages and articular chondrocytes [12, 20]. Previous studies have demonstrated that tomatidine can induce apoptosis effects in osteosarcoma, lung cancer, and breast cancer [11, 21–23]. Tomatidine also improves nonalcoholic fatty liver disease in mice with high-fat diet-induced obesity [14]. Furthermore, tomatidine ameliorates airway hyperresponsiveness and suppresses Th2 cell activity in a murine model of asthma [13]. Here, we investigated whether tomatidine improves inflammation in the lungs of a murine model of ALI.

The development of ALI is mainly due to an acute and unbalanced inflammatory response that exacerbates damage to epithelial or endothelial cells, causing excess protein fluid in the plasma into the alveoli, causing edema of the alveoli and interstitial cells and excessive inflammatory immune cell infiltration into the lungs [1, 3]. When bacteria invade, they release LPS, causing inflammation and fever [7]. In addition, LPS and other inflammatory substances stimulate and destroy the microvascular barrier of the alveoli and destroy the tight junctions between epithelial or endothelial cells [3]. The damaged barrier between cells will lose the ability to protect the integrity of alveolar and airway function, and have a decreased ability to remove excess body fluid and infectious microorganisms, causing pulmonary edema [24]. Thus, improving inflammation in lung epithelial cells is a potential treatment approach for ALI.

Neutrophils have the ability to swallow invading bacteria to reduce lung injury [25]. However, activated neutrophils release excessive inflammatory mediators, causing severe oxidative damage [26]. Tomatidine can effectively attenuate neutrophil infiltration into the BALF and lungs and contribute to reducing lung inflammation. MPO is an enzyme that is found mainly in neutrophils [27]. MPO activity is also the main marker of neutrophils and represents an indicator of neutrophil infiltration in the lungs [28]. In the present study, tomatidine significantly inhibited the MPO activity, confirming that tomatidine can inhibit LPS-induced lung injury and attenuate lung inflammation caused by neutrophil infiltration.

In ALI patients, inflammatory cytokines are increased and cause fever and sepsis [3]. LPS can stimulate lung macrophages or lung epithelial cells to secrete inflammatory cytokines, inducing lung tissue damage and leading to pulmonary edema [2, 29]. Our research confirmed that tomatidine alleviates inflammatory cytokine secretions by reducing LPS-induced lung injury in mice. Subsequently, gene expression in the lung demonstrated that, compared to mice with lung injury, tomatidine attenuates the gene expression of inflammatory mediators (ICAM-1, COX-2, and iNOS). Using LPS-stimulated lung epithelial cells for in vitro experiments, tomatidine can effectively reduce inflammation-associated cytokine and chemokine productions. Thus, tomatidine contributes to reducing the inflammatory response in LPS-induced lung injury.

LPS- or inflammatory cytokine-stimulated lung epithelial cells attract immune cells to infiltrate the lungs and induce oxidative stress, thereby destroying lung cells and weakening lung function [17, 30]. Tomatidine reduces MCP-1 and IL-8 levels, inhibiting the migration of macrophages and neutrophils to infiltrate the lungs [30]. MDA activity is a common marker of oxidative stress [3]. GHS and SOD are also mainly antioxidant enzymes that regulate oxidative stress in the lungs [31]. In the current study, tomatidine significantly reduced MDA levels and promoted GSH and SOD production in the lungs of mice with ALI. In addition, cells stimulated by oxidative stress and Nrf2 translocate into the nucleus to induce HO-1 expression [32]. Tomatidine significantly increases nuclear Nrf2, thereby inducing HO-1 expression and enhancing the protective effect against oxidative stress. Therefore, tomatidine has the ability to resist oxidative stress and can maintain lung function in mice with lung injury.

LPS stimulates NF-*κ*B pathway activation, causing I*κ*B phosphorylation and NF-*κ*B release into the nucleus; therefore, it would promote the gene expression of oxidative and inflammatory mediators [33]. In addition, the activity of the MAPK pathway can promote the transcription of proinflammatory genes, aggravating lung injury and sepsis in ALI mice [34]. MAPK inhibitor has been shown to reduce the inflammatory response and suppress neutrophil infiltration in ALI mice [35]. Furthermore, many flavonoids, including resveratrol, phloretin, and lutein, can alleviate inflammation in mice with lung injury by attenuating the NF-*κ*B and MAPK pathways [16, 36, 37]. Tomatidine could significantly inhibit the MAPK and NF-*κ*B pathways and reduce ICAM-1 expression, thereby suppressing neutrophil infiltration and reducing the secretion of inflammatory mediators in mice with ALI.

In conclusion, tomatidine can effectively ameliorate inflammation and oxidative stress in LPS-induced lung injury, mainly through inhibition of the NF-*κ*B and MAPK pathways. These results provide experimental evidence that tomatidine is beneficial in improving ALI.

## Data Availability

The data used to support the findings of this study are included within the article.
